# Protocol for single-molecule imaging of transcription and epigenetic factors in human neural stem cell-derived neurons

**DOI:** 10.1016/j.xpro.2024.103432

**Published:** 2024-11-01

**Authors:** Yuri Atsumi, Nobuhiko Yamamoto, Noriyuki Sugo

**Affiliations:** 1 Graduate School of Frontier Biosciences, Osaka University, Suita, Osaka 565-0871, Japan; 2 Institute of Neurological and Psychiatric Disorders, Shenzhen Bay Laboratory, Shenzhen, Guangdong 518132, China

**Keywords:** Single-molecule Assays, Cell culture, Microscopy, Neuroscience

## Abstract

Single-molecule imaging (SMI) is a powerful approach to quantify the spatiotemporal dynamics of transcription in living cells. Here, we describe a protocol of SMI for transcription and epigenetic factors in human cortical neurons derived from embryonic stem cells or induced pluripotent stem cells. Specifically, we detail the procedures for neural stem cell culture, gene transfer, microscopy, and data analysis. This protocol can be applied to the study of transcription dynamics in response to various cellular stimuli.

For complete details on the use and execution of this protocol, please refer to Atsumi et al.^1^

## Highlights

• Long-term culture of human ES cell-derived cortical neurons from neural stem cells
• Single-molecule imaging of transcription factors under HILO microscope
• Simultaneous dual-color single-molecule imaging of transcription and epigenetic factors
• Quantitative analysis of spatiotemporal properties of single-molecule spots

## Abstract (editor highlights)

Publisher’s note: Undertaking any experimental protocol requires adherence to local institutional guidelines for laboratory safety and ethics.

## Graphical abstract

**Figure:**
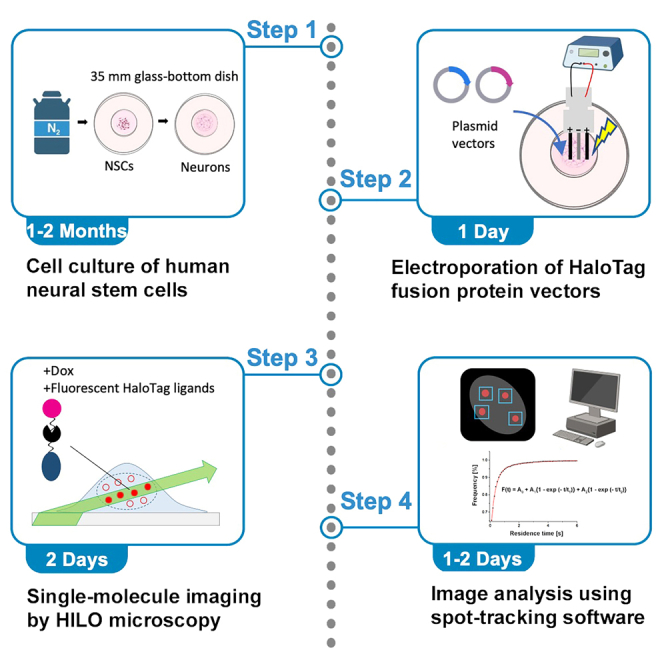

## Before you begin

**Figure 1:**
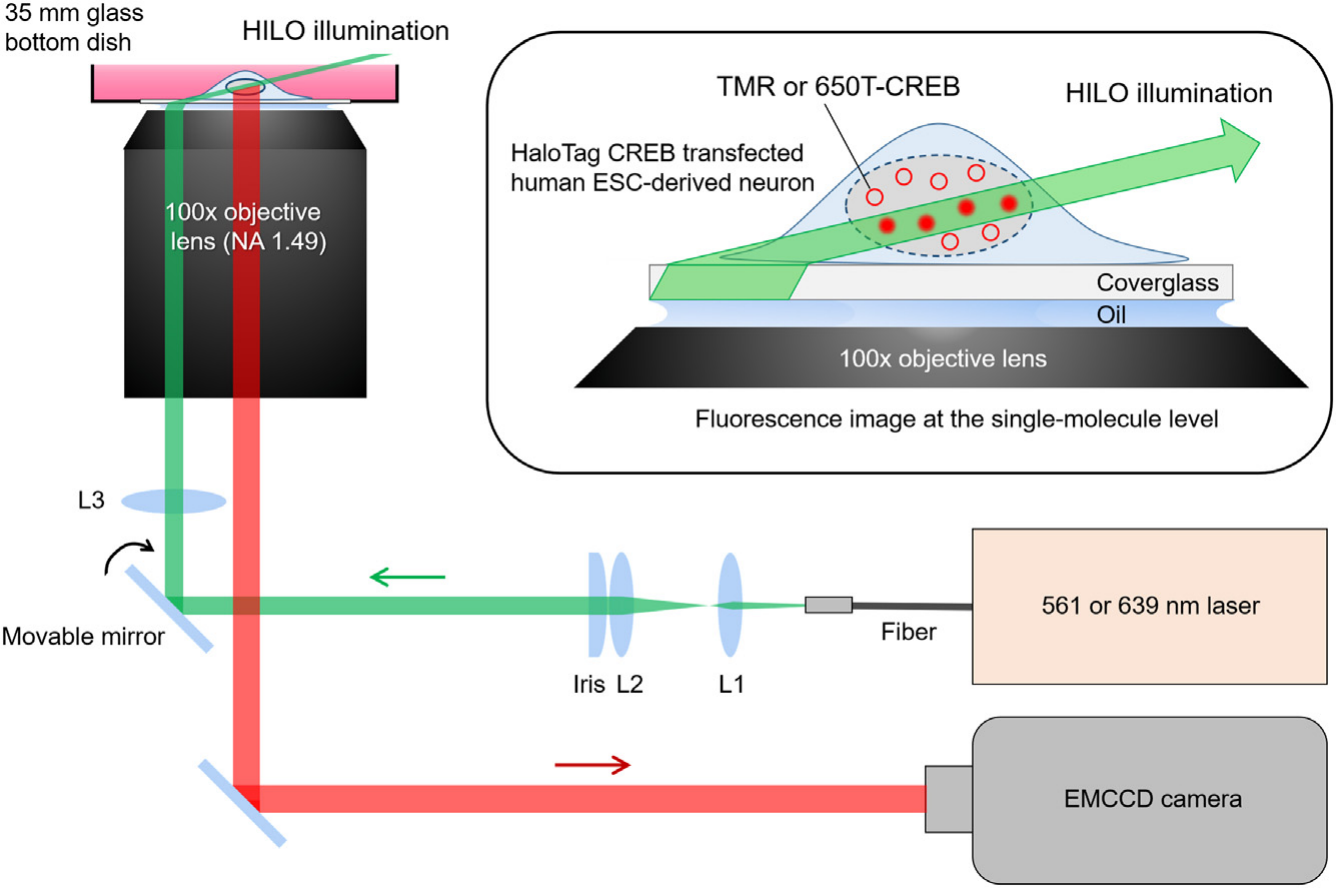
Schematic of HILO microscopy An incident laser beam, controlled by a movable mirror, is positioned to propagate near the edge of the 100× oil-immersion objective lens, and the resulting HILO illuminates nuclei in a single cell cultured on a glass-bottom dish. Fluorescent signals with low background noise from the fluorescence-tagged proteins through the same objective lens are captured by an EMCCD camera.

Protein-DNA and protein-protein interactions are important issues in understanding transcription mechanisms.^2^^,^^3^^,^^4^ Highly inclined and laminated optical sheet (HILO) microscopy in combination with HaloTag and/or SNAP-tag proteins and their fluorescent ligands is an excellent experimental approach to observe the spatiotemporal dynamics of proteins, such as transcription and epigenetic factors in the nucleus, in living cells at the single-molecule level (Figure 1).^5^^,^^6^^,^^7^^,^^8^^,^^9^^,^^10^^,^^11^ Here we demonstrate a detailed method of single-molecule imaging (SMI) to reveal the behavior of the transcription factor cAMP response element binding protein (CREB) and the histone acetyltransferase CREB binding protein (CBP)^12^^,^^13^^,^^14^^,^^15^^,^^16^ in human neural stem cell (NSC)-derived neurons.^17^^,^^18^^,^^19^ This protocol can be used for a variety of primary cells and cell lines and can be applied to the studies of diverse intracellular proteins including nuclear proteins.

### Institutional permissions

Undertaking this experimental protocol requires adherence to local institutional guidelines for laboratory safety and ethics. All experiments using human NSCs were conducted with the approval of the Osaka University Ethical Committees.

### Design and construction of plasmid vectors expressing HaloTag or SNAP-tag-proteins under a tetracycline-inducible promoter

This protocol uses plasmid vectors that contain genes tagged with HaloTag and SNAP-tag under a tetracycline-inducible promoter (Tet-On).^8^^,^^20^ The Tet-on system allows to control an appropriate expression level for SMI by optimizing the amount of doxycycline (Dox) added to the culture medium. In addition, these distinct tagging systems can allow usage of different color fluorophores simultaneously, making it possible to observe interactions between multiple proteins. The users can select a tag and a vector containing a gene of interest (GOI). After plasmid construction, the maxi-prep purification kit can be used to prepare a large amount of plasmid DNA for electroporation.

***Note:*** HaloTag and SNAP-tag are useful for one-to-one labeling with specific ligands conjugated with fluorescent molecules, which have strong fluorescence intensities and lifetimes. The HaloTag system is recommended as the first choice rather than the SNAP-tag system because the HaloTag ligand has a faster reaction kinetics than the SNAP-tag ligand.^21^ The intracellular localization and expression of the tagged proteins should be confirmed by comparing with those of the endogenous proteins in appropriate cells (see details in Figures S2C-F in Atsumi et al^1^).

***Note:*** A plasmid containing CMV (or select an appropriate promoter)-driven rtTA and TRE-driven GOI is necessary for the Tet-On system (e.g. pTet-On Advance/TRE-Tight HaloTag-CREB). The backbone plasmid is commercially available (e.g. pTetOne Vector, Clontech). In the case of dual-color SMI, TRE-driven plasmid is sufficient to drive the second GOI (e.g. pTRE-SNAP-tag-CBP).

***Note:*** Co-transfection with a plasmid encoding fluorescent protein is useful to find HaloTag fusion protein expressing cells. pTα1-EGFP plasmid is suitable to label neuronal cells.^22^

**CRITICAL:** It is important to obtain highly purified plasmids with a transfection-grade plasmid purification kit.

## Key resources table

**Table:** 

REAGENT or RESOURCE	SOURCE	IDENTIFIER
**Antibodies**		

Anti-MAP2, mouse monoclonal (1:500 dilution)	Sigma	Cat#M1406; RRID: AB_477171
Anti-BRN2, goat polyclonal (1:400 dilution)	Santa Cruz	Cat#sc-6029; RRID: AB_2167385
Anti-CTIP2, rat monoclonal (1:500 dilution)	Abcam	Cat#ab18465; RRID: AB_2064130
Anti-TBR1, rabbit polyclonal (1:500 dilution)	Abcam	Cat#ab31940; RRID: AB_2200219

**Chemicals, peptides, and recombinant proteins**		

Hanks' balanced salt solution	Gibco	Cat#14175-095
DMSO	Millipore	Cat#67-68-5
Trypan blue solution	Wako	Cat#207-17081
Poly-L-ornithine	Sigma	Cat#P3655
Matrigel	Corning	Cat#356234
DMEM/F12	Gibco	Cat#11320-033
GlutaMAX	Gibco	Cat#355050-061
N2 supplement	Gibco	Cat#17502-048
Non-essential amino acids	Gibco	Cat#11140-050
Sodium pyruvate	Gibco	Cat#11360-070
Bovine serum albumin (BSA)	Gibco	Cat#15260-037
β-mercaptoethanol	Sigma	Cat#M3148-25ML
Penicillin/streptomycin	Gibco	Cat#15070-063
Neurobasal	Gibco	Cat#21103-049
B27 supplement without vitamin A	Gibco	Cat#12587-010
L-Glutamine	Nacalai	Cat#16919-42
Doxycycline (Dox)	Clontech	Cat#631311
TMR-conjugated HaloTag ligand	Promega	Cat#G2991
SaraFluor 650T-conjugated HaloTag ligand	Goryo Chemical	Cat#A308-01
Janelia Fluor 646-conjugated HaloTag ligand	Promega	Cat# GA1120
647SiR-conjugated SNAP-tag ligand (SNAP-Cell 647-SiR)	NEB	Cat# S9102S
TetraSpeck fluorescent microspheres	Invitrogen	Cat# T7284

**Critical commercial assays**		

Plasmid maxi kit	QIAGEN	Cat#12162 or 12363
CompactPrep plasmid midi kit	QIAGEN	Cat#12843

**Recombinant DNA**		

pTα1-EGFP	Hatanaka et al.^22^	N/A
pTet-On Advance/TRE-Tight HaloTag-CREB	Kitagawa et al.^8^	N/A
pTRE-SNAP-tag-CBP	Atsumi et al.^1^	N/A

**Software and algorithms**		

NIS Elements Advanced Research	Nikon	https://www.nikoninstruments.com/Products/Software; RRID:SCR_014329
Andor SOLIS	Andor Technology	https://andor.oxinst.jp/products/solis-software/
ImageJ	NIH, USA	https://imagej.net/ij/; RRID:SCR_003070
Particle Track and Analysis (PTA) (A new version, PTA2, is also available)	Sugo et al.^7^	https://github.com/arayoshipta/projectPTAj https://github.com/arayoshipta/PTA2
Origin 2021	OriginLab	http://www.originlab.com/index.aspx?go=PRODUCTS/Origin; RRID:SCR_014212

**Other**		

35 mm glass bottom dish (CELLview sterile cell culture dishes with glass bottom)	Greiner Bio-One	Cat#627860
35 mm Petri dish	Falcon	Cat#351008
Nalgene general long-term storage cryogenic tube	Thermo Scientific	Cat#5000-0020
Disposable Pasteur pipet (9 inch)	Fisher Scientific	Cat#13-678-20C
Automated cell counter	Logos Biosystems	Luna
Centrifuge	Thermo Scientific	Sorvall ST 8FR
Luna cell counting slide	Logos Biosystems	Cat#NC1765657
Electroporator	BEX	CUY21EX
Plate electrodes	BEX	LF513-5
Inverted microscope	Nikon	Ti-E
NeuroMag	OZ Biosciences	Cat# NM50500
Super magnetic plate	OZ Biosciences	Cat# MF10000
10x objective lens	Nikon	Plan Fluor 10x/0.30
100x oil-immersion lens	Nikon	Apo TIRF 100x/1.49 oil
Stage top incubator	TOKAI HIT	INU-NI-F1
CCD camera	Photometrics	CoolSNAP HQ2
EMCCD camera	Andor Technology	iXon897
Refrigerated/heating circulator	Julabo	F12-ED
Mercury lamp	Nikon	Intensilight C-HGFIE
561 nm laser	Coherent	Sapphire, 20 mW
639 nm laser	Coherent	Cube, 40 mW
Lens, L1 (f = 10 mm)	Thorlabs	AL-1210-A
Lens, L2 (f = 100 mm)	Thorlabs	AC254-100-A-ML
Lens, L3 (f = 500 mm)	Thorlabs	AC254-500-A-ML
Iris	Thorlabs	SM1D12C
Image splitting module (W-VIEW GEMINI)	Hamamatsu Photonics	Cat#A12801-01
640 nm long-pass dichroic mirror (>640 nm)	Semrock	Cat#FF640-FDi01-25×36
Bypass filter for 593 nm (573–613 nm)	Semrock	Cat#FF01-593/40-25
Bypass filter for 692 nm (672–712 nm)	Semrock	Cat#FF01-692/40-25
Immersion oil for microscopy	Nikon	Type F2
FoilCover	PeCon	FoilCover-set for 35 mm Petri dishes

## Materials and equipment

Prepare the following solutions.

**Table:** DDM/B27

Reagent	Final concentration	Amount
DMEM/F12	N/A	46.2 mL
GlutaMAX (100 x)	1x	0.5 mL
N2 supplement (100 x)	1x	0.5 mL
non-essential amino acids (100 x)	1x	0.5 mL
B27 without vitamin A (50 x)	1x	1 mL
sodium pyruvate (100 x)	1x	0.5 mL
bovine serum albumin (BSA, 7.5%)	0.05%	0.33 mL
penicillin/streptomycin (100x)	1x	0.5 mL
10-fold diluted β-mercaptoethanol (1.43 M)	0.1 mM	3.5 μL
**Total**	**N/A**	**50 mL**

Store at 4°C for up to one week.

**Table:** NeuroBasal/B27

Reagent	Final concentration	Amount
Neurobasal	N/A	48 mL
B27 without vitamin A (50 x)	1x	1 mL
L-glutamine (200 mM)	2 mM	0.5 mL
penicillin/streptomycin (100x)	1x	0.5 mL
**Total**	**N/A**	**50 mL**

Store at 4°C for up to one week.

**Table:** N2/B27

Reagent	Final concentration	Amount
DDM/B27	N/A	25 mL
NeuroBasal/B27	N/A	25 mL
**Total**	**N/A**	**50 mL**

Store at 4°C for up to one week.

***Note:*** The above volumes are sufficient to generate and maintain a small scale of cultures (about 10 dishes).

### Matrigel

Make 20 μL aliquots of Matrigel on ice and store them at −80°C for at least one year.

### Poly-L-ornithine solution

Dissolve 100 mg of poly-L-ornithine in 10 mL of 0.15 M borate buffer (pH 8.5). Make aliquots and store them at −20°C for at least one year.

### Dox solution

Dissolve 1 mg of Dox in 10 mL Milli-Q water (100 μg/mL). Make aliquots and store them at −20°C for at least one year.

### Fluorescent ligand solution

Prepare 50 μM stock solutions of HaloTag ligand conjugated with TMR, SaraFluor 650T or Janelia Fluor 646 dyes by dissolving with DMSO. For dual-color simultaneous SMI, prepare stock solutions of 50 μM TMR-conjugated HaloTag and 50 μM 647SiR-conjugated SNAP-tag ligand by dissolving with DMSO. Store them at −20°C for at least one year.

### Microscopy

**Figure 2:**
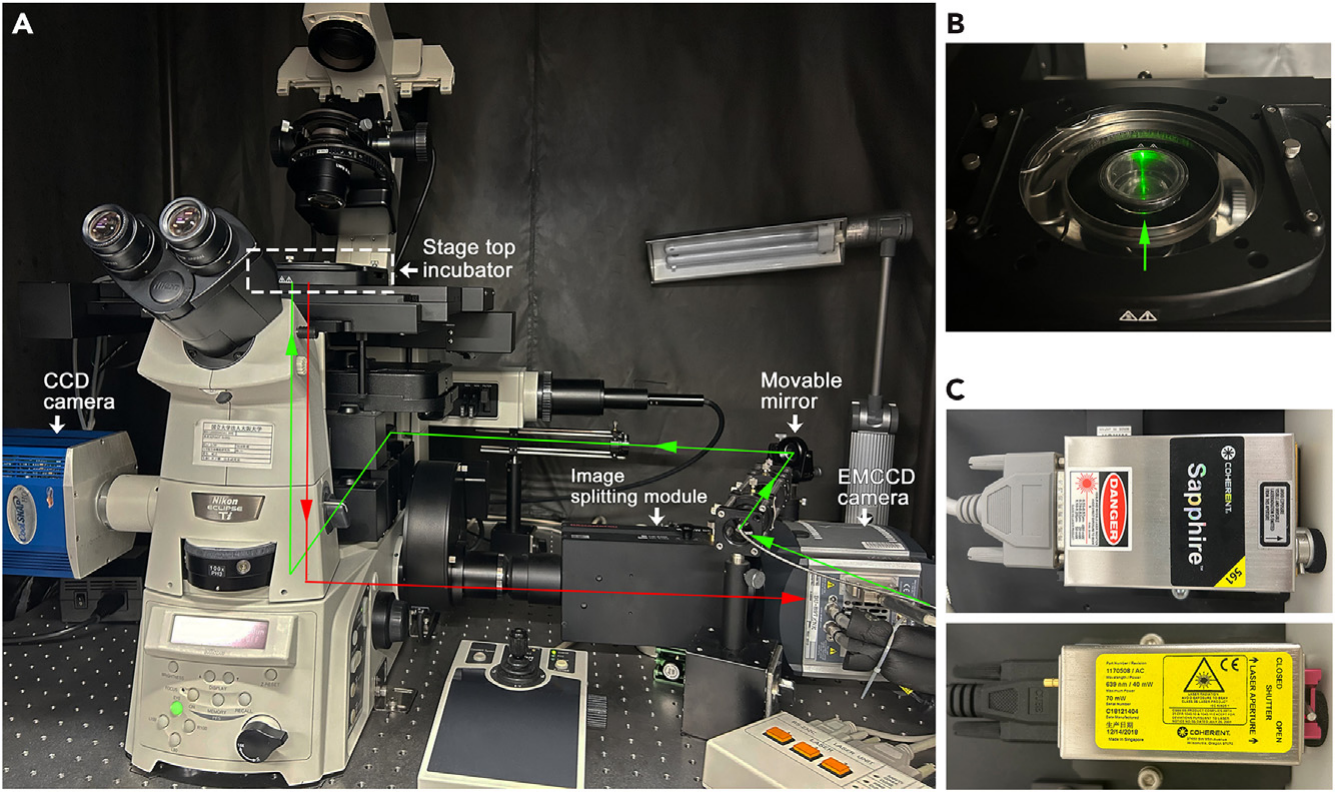
Microscopy for SMI and simultaneous dual-color SMI (A) Setup for SMI consisting of laser system, HILO illumination and EMCCD camera. Image splitting module for simultaneous dual-color SMI is mounted in front of the EMCCD camera. (B) Top view of the culture dish on the stage top incubator indicated by dotted square. (C) HILO illumination is emitted by 561 nm and 639 nm lasers.

Setup an inverted microscope (e.g., Ti-E, Nikon) equipped with a 100x oil-immersion objective lens (NA 1.49), a stage top incubator (TOKAI HIT), a monochrome CCD camera (Photometrics), and an EMCCD camera (Andor Technology) (Figures 1 and 2). Select appropriate lasers for HILO illumination. We use 561 nm and 639 nm lasers (Coherent, 20 mW and 40 mW, respectively) to detect TMR and SaraFluor 650T, respectively. The illumination beam is controlled by a movable mirror to propagate near the edge of the objective lens. Fluorescent signals through the objective are captured by an EMCCD camera cooled by a recirculating water cooler (Julabo, set to 17°C). A monochrome CCD camera and a mercury lamp (Nikon) are utilized to find GFP-positive cells at a lower magnification (e.g., 10x). Install NIS element Advanced Research (Nikon) and Solis software (Andor Technology) on a personal computer connected to the microscope and cameras.

For dual-color simultaneous SMI, an image splitting module (W-VIEW GEMINI, Hamamatsu Photonics) is mounted just before the EMCCD camera (Figures 2A–2C). This optical system enables to provide one pair of dual wavelength images separated by a dichroic mirror onto a single camera. Select a dichroic mirror with appropriate filters.

## Step-by-step method details

### Human NSC culture

**Timing: One to two months before SMI**

NSCs can be generated from human ESCs or iPSCs according to previously established protocols.^18^^,^^19^^,^^23^ The initially obtained NSCs (several million cells) can be amplified by a single passage. As a result, 50 to 100 aliquots (approximately 500,000 cells, 0.5 mL) will be obtained and can be stored in liquid nitrogen for at least one year.^17^^,^^23^ This amplification enables to use homogeneous NSCs for experiments. The most important feature of our culture method is the plating of a small amount of cells (10,000 to 20,000 cells) in the central portion of the glass bottom dish. Such a small-scale culture is sufficient for imaging experiments. Moreover, only a small amount of culture medium is sufficient to maintain the cultures. NSCs continue to proliferate to some extent, but gradually postmitotic cortical cells emerge, and thin-layer cultures can be maintained for a few months.

Plating NSCs on the glass-bottom dishes to make monolayer cell cultures.

**Figure 3:**
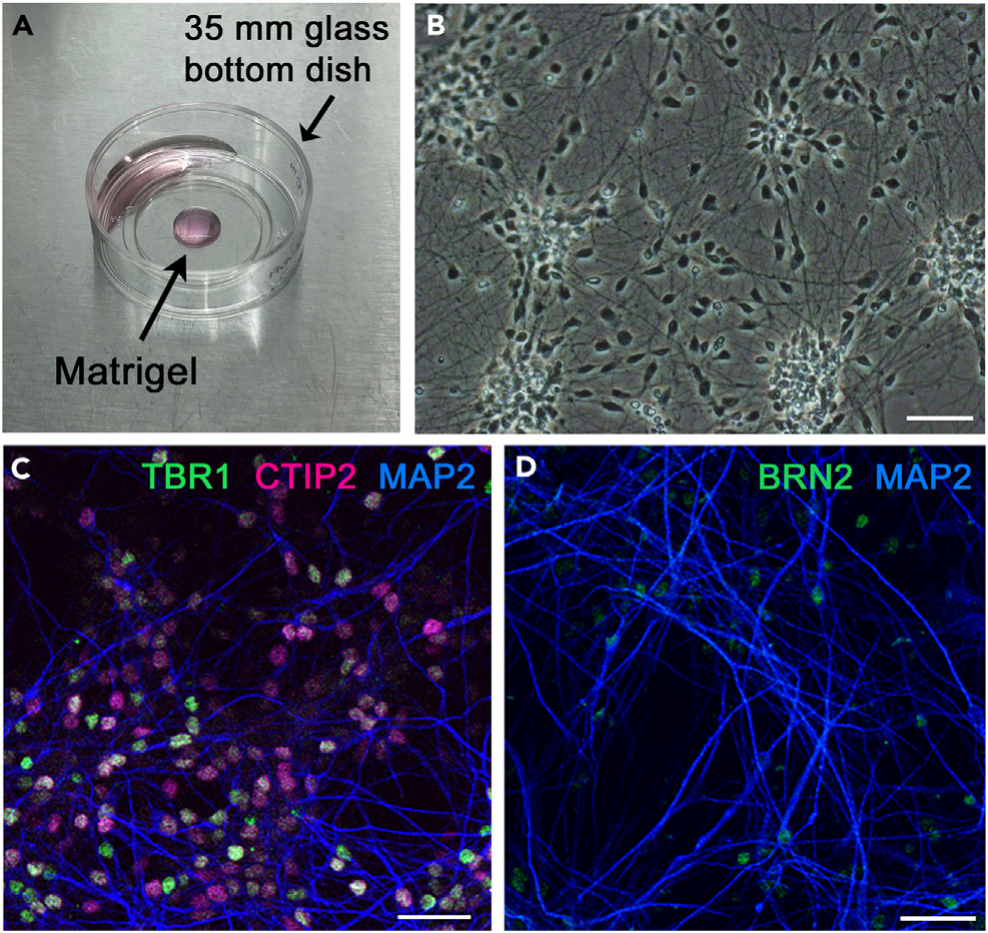
Culture method for human NSCs and neuronal differentiation (A) Matrigel coating on the glass-bottom dish. (B) Phase-contrast picture of NSC culture. Scale bar: 50 μm. (C and D) Immunocytochemical analysis of cultured cells with antibodies. Scale bar: 50 μm. Figures adapted from Atsumi et al.^1^
  c. Keep these dishes at 25°C for more than 3 h.
3. Thaw NSCs stored in liquid nitrogen.
  a. Take one cryotube containing NSC cells (approximately 500,000 in 0.5 mL aliquot) from the liquid nitrogen tank, and keep the cryotube in the liquid nitrogen container or on dry ice before use.
  b. Add 5 mL of warm N2/B27 to a 15-mL centrifugation tube.
  c. Place the cryotube in a 37°C water bath.
  d. Immediately after thawing, add the cell suspension (0.5 mL) to the 15 mL tube with a fire-polished Pasteur pipette, and mix well by inverting the tube.
4. Make cell suspension of NSCs.
  a. Centrifuge the 15 mL tube for 3 min (1000 rpm or 190 *g*), and discard the supernatant carefully.
  b. Resuspend cells with 200–300 μL of N2/B27 with a fire-polish Pasteur pipette.
  c. Count the number of live cells with trypan blue. The cell density (trypan blue-negative cells) will be approximately 500,000–1000,000 cells/mL.
5. Plate NSCs on poly-L-ornithine- and Matrigel-coated dishes.
  a. Discard the Matrigel solution on the glass bottom dishes (see step 2).
  b. Add the cell suspension (10–20 μL) at exactly the same location as Matrigel-coated region, so that 10,000 to 20,000 cells can be plated.
  c. A few hours after confirming cell attachment to the bottom glass, add 300–500 μL of N2/B27 to the droplet containing NSCs.

***Note:*** Add the cell suspension before Matrigel coating dries out. Confirm that cells are confluent before step 5c. If not, further add a small volume (5–10 μL) of cell suspension. Thus, more than ten dishes on which NSCs are plated will be obtained from one cryotube.

1. Prepare poly-L-ornithine-coated culture dishes.
  a. Dilute the poly-L-ornithine stock with Milli-Q water (100-fold dilution, final concentration: 0.1 mg/mL).
  b. Add 100–200 μL of poly-L-ornithine solution to the glass area of the 35 mm glass bottom dishes, and incubate the dishes for more than 1 h at 25°C.
  c. Remove the poly-L-ornithine solution and wash 2 to 3 times with Milli-Q water.
  d. Dry the dishes for roughly 30 min under a laminar flow cabinet.
2. Prepare poly-L-ornithine-coated and Matrigel-coated culture dishes.
  a. Dilute the Matrigel stock with chilled N2/B27 medium (40- to 80-fold dilution). Since Matrigel solution tends to gel at 25°C, keep it on ice until just before adding to the dish.
  b. Place 20–25 μL of the diluted Matrigel on the center of the poly-L-ornithine coated dishes (see Figure 3A). Add 300–400 μL of N2/B27 to the surrounding of the bottom glasses, so that the Matrigel solution does not dry out (Figure 3A).

6. Add and exchange the culture medium with fresh N2/B27 before the culture medium is reduced or acidic. The volume of the culture medium is 1–2 mL.
7. Check whether cortical cells are generated from NSCs after one to two months in culture (Figure 3B). For this, perform immunostaining with antibodies against MAP2, BRN2 (for upper layer cells), CTIP2 (for layer 5 cells), and TBR1 (for layer 6 cells) (Figures 3C and 3D).^18^^,^^19^

### *In vitro* electroporation

**Timing: 3–7 days prior to SMI**

To introduce plasmids into cultured neurons, electroporation can be used.^8^^,^^20^ The transfection method for the HaloTag-CREB plasmid is shown here. The following procedure is performed in a laminar flow cabinet.

**Figure 4:**
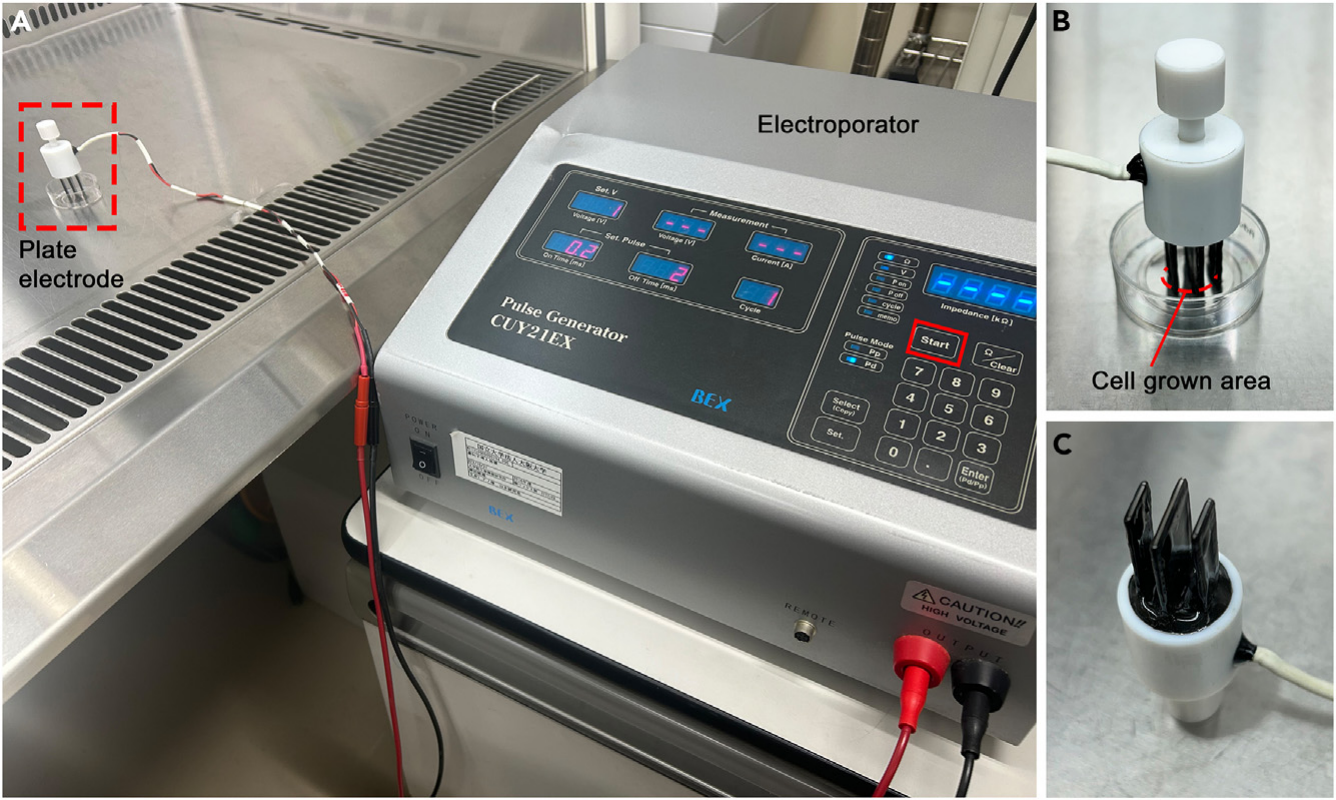
Electroporation into cultured neurons with plate electrodes (A) Set-up for electroporation. (B and C) Plate electrodes and its arrangement on the glass-bottom dish.
10. Set the parameters on the electroporator to deliver electric pulses composed of one 275 V pulse of 7 ms duration and five 20 V pulses of 50 ms duration at 50 ms intervals.
11. Prepare plasmid solution in a 1.5 mL Eppendorf tube.

***Note:*** Prepare a sufficient volume of plasmid solution to cover the cells. The final plasmid concentration should not exceed 1.0 μg/μL. In our case, the plasmid solution is composed of 50 μg pTα1-EGFP and 100 μg pTet-On Advance/TRE-Tight HaloTag-CREB in 200 μL PBS (troubleshooting 1).

8. Sterilize the plate electrodes with ethanol.
  a. Immerse the plate electrodes in 70% ethanol in a 35 mm petri dish for 15 min.
  b. Put the plate electrodes in an empty petri dish to dry.
9. Connect the electroporator to the sterilized plate electrodes (Figure 4A).

12. Remove the culture medium from the dish and keep it in a 1.5 mL tube.
13. Add 200 μL plasmid solution in the center of the culture dish.
14. Put the plate electrodes gently on the cultured cells (Figures 4B and 4C).
15. Press the “Start” button on the electroporator to deliver electric pulses.

***Note:*** Ensure that the current is 0.7–1.1A. To increase the number of transfected cells, electroporation can be performed twice on the same culture. The second electroporation can be applied by rotating the electrodes by 90 degrees.

16. Remove the plate electrodes carefully from the cultured cells and put them in a PBS-containing dish until the next culture dish is electroporated.

***Note:*** After delivering electric pulses, cell debris would adhere to the electrodes. Such debris can be removed by soaking the electrodes in PBS.

17. The plasmid solution can be reused for 4 to 6 culture dishes.
18. Wash the cells with 1–2 mL PBS.
19. Add the stored medium (see step 12) to the cells and maintain the culture at a CO_2_ incubator.
20. The electroporation can be repeated with the same plasmid solution.

***Note:*** After the electroporation experiment, clean the plate electrodes. Wash the plate electrodes with tap water. Dry it completely. Polish the electrodes with a fine sandpaper to remove rust from them; otherwise, the transfection efficiency will be drastically reduced.

### Induction of HaloTag-CREB expression

**Timing: 17–24 h prior to SMI**

21. Add 1 μL of 10-fold dilution of the Dox solution to 1 mL culture medium (final concentration: 10 ng/mL).
22. Stir the culture dish or pipet gently the culture medium.
23. Maintain the Dox-treated cultures in a CO_2_ incubator for 17–24 h.

**CRITICAL:** The Dox concentration should be optimized according to experimental conditions. For example, HaloTag-CREB expression is induced at 0.05 ng/mL Dox in mouse cortical neurons, while it is induced at 10 ng/mL Dox in human NSCs.

### Labeling of fluorescent HaloTag ligands

**Timing: 30 min prior to SMI**

24. To make HaloTag ligand-containing medium, add 1 μL of 5-fold diluted TMR-, Janelia Fluor 646- or SaraFluor 650T-conjugated HaloTag ligand solution to 1 mL DMEM/F-12 (final concentration: 10 nM).
25. Remove the culture medium and keep it in a 1.5 mL tube.
26. Add 1 mL of DMEM/F-12 containing fluorescent HaloTag ligand.
27. Maintain the HaloTag ligand-treated culture at 37°C in a CO_2_ incubator for 15 min.
28. Remove the ligand-containing medium and return to the original medium.

***Note:*** There are several cell membrane-permeable fluorescent ligands even at similar wavelengths. An appropriate one for the experiment should be chosen. The same ligand should be used for a series of experiments.

### Single-molecule imaging

**Timing: 3–6 h**

29. At least 1 h prior to SMI, activate the following equipment and software (see materials and equipment).
  a. Fill the stage top incubator (Tokai Hit) with Milli-Q water and supply 5% CO_2_ gas (95% air) to the incubator.
  b. Turn on the inverted microscope, the mercury lamp, the monochrome CCD camera, the EMCCD camera, the computer, the recirculating water cooler for the EMCCD camera, the laser and the stage top incubator (set to the following heating parameter: top heater 38.0°C, bath heater 36.5°C, stage heater 38.0°C, lens heater 37.0°C).
  c. Open the NIS Elements software and select “Roper Scientific” to capture fluorescent images using the monochrome CCD camera.
  d. Open the Solis software linked to the EMCCD camera. Wait until the EMCCD camera has cooled to −90°C.

***Note:*** Wait at least 1 h after power-up for all instruments to reduce drift during SMI. It is also important to know the extent of drift using fluorescent beads.

**Figure 5:**
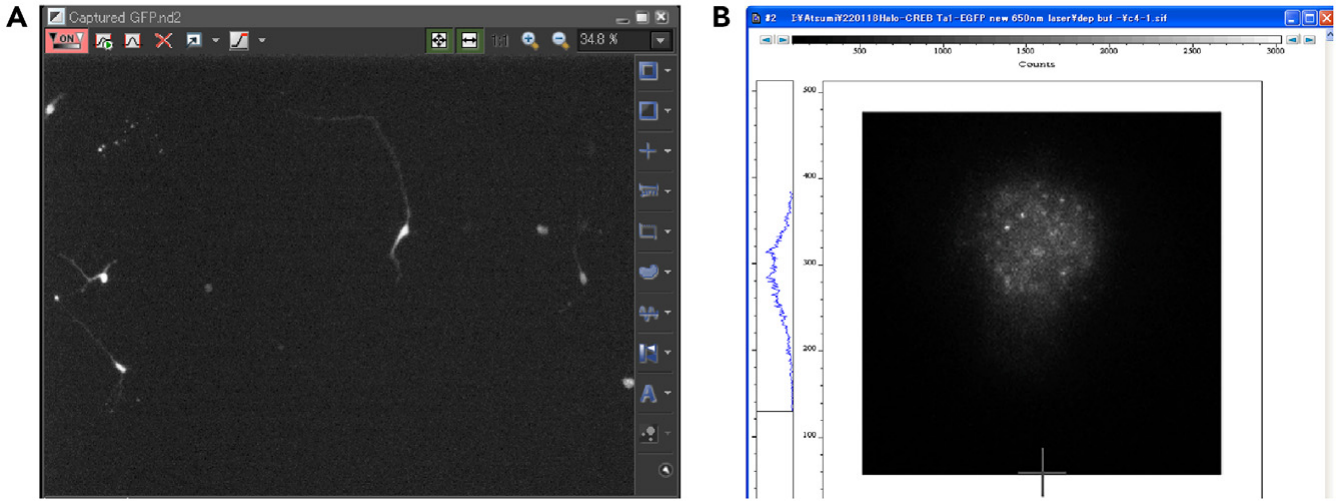
Data acquisition of SMI using microscopy software (A) Search for EGFP-positive cells using CCD camera and NIS element software with a lower magnification lens (x10). (B) Subsequently, switch to a higher magnification lens (x100) and obtain imaging data of fluorescent-tagged CREB spots in the EGFP-positive cell using EMCCD camera and Solis software.
37. Lower the position of the lower magnification lens, and switch to the 100x oil-immersion lens.

***Note:*** If you cannot find EGFP-positive cells with 100x oil-immersion lens, switch to 10x lens to find transfected cells again.

30. Set the parameters on Solis software.
  a. Exposure time: 0.1 s.
  b. Kinetics Series Length: 1200 (2-min observation).
  c. Electron Multiplier Gain Level: 300.
  d. ROI: 256 × 256 pixels.
  e. Other parameters are the same as the default.
31. Place one drop of the immersion oil on 100x oil-immersion objective lens. Lower the lens position.
32. Mount the sample on the stage top incubator maintained at 37°C in a humidified atmosphere (5% CO_2_, 95% air).
33. Maintain the sample for 30 min before starting the observation.
34. Switch to a lower magnification lens (e.g., 10x).
35. Click the “Live” button in NIS elements and turn on epi-fluorescence illumination of the mercury lamp (excitation, 488 nm).
36. Search for EGFP-positive cells in “Live” mode of NIS element using joystick with a coarse mode and adjust the focus. EGFP-positive cells can be observed with 0.1 s exposure time. Capture the green fluorescent images at a lower magnification to record the observed area (Figure 5A).

38. Adjust XY stage and the focus using joystick with a fine or extra fine mode. Ensure that oil is spread between the glass bottom and the lens.
39. Turn off the lamp illumination light (488 nm).
40. Switch to EMCCD camera and Solis software, and turn on laser. TMR is excited by 561 nm laser, while SaraFluor 650T is excited by 639 nm laser.

***Note:*** Use Solis software for SMI to obtain higher temporal resolution images.

41. Click the “Video” button in Solis software and check whether fluorescent-tagged CREB spots emerge in the nuclei by HILO illumination (Figure 5B and Methods video S1 left). When you hardly find CREB spots, find other EGFP-positive cells by epifluorescence illumination with appropriate filter and CCD camera with NIS Element software (troubleshooting 2).
42. Tune the illumination angle of the movable mirrors and change the laser power manually, to attain good image quality.
43. Click the “Take Signal” in Solis software to obtain imaging data (troubleshooting 3).

***Note:*** To reduce focus drift during capture, we recommend that you use the Nikon Perfect Focus Systems.

44. After 2-min imaging, turn off the laser and save the imaging data in SIF (Andor related image format).

***Note:*** Because the fluorescence of the tagged proteins excited by laser is attenuated by photobleaching, keep the laser off when not observing. During 2-min observation bleaching will often take place. However, after a few minutes, you will see bright spots in the same nucleus, and collect additional images.

45. Take EGFP images with Solis software at the 100x oil-immersion lens to memorize the morphology of the nuclei.
46. Switch to the monochrome camera and NIS Element software.
47. Repeat steps 35–46. You can obtain SMI images from ∼10 cells for 1 h.

***Note:*** To define a single molecule, examine the photobleaching curve of fluorescent spots after fixation. If the spot is a single molecule, the curve should show a one-stepwise reduction of the fluorescence intensity (Figure 7D). It would be better to perform negative control experiments such as ligand alone, HaloTag alone vector, SNAP-tag alone vector, and without Doxycycline.

***Note:*** Compare the photobleaching time and the residence time of fluorescent-tagged spots to check if the photobleaching affects the residence time. In our case, the time constant for the photobleaching time was much longer than the long residence time.^7^ The laser should be irradiated intermittently if it is not longer than the residence time.^9^^,^^11^

Methods video S1. Example of SMI of CREB and the spot analysis with PTA, related to step 41

### Simultaneous dual-color SMI

**Timing: 3–6 h**

**Figure 6:**
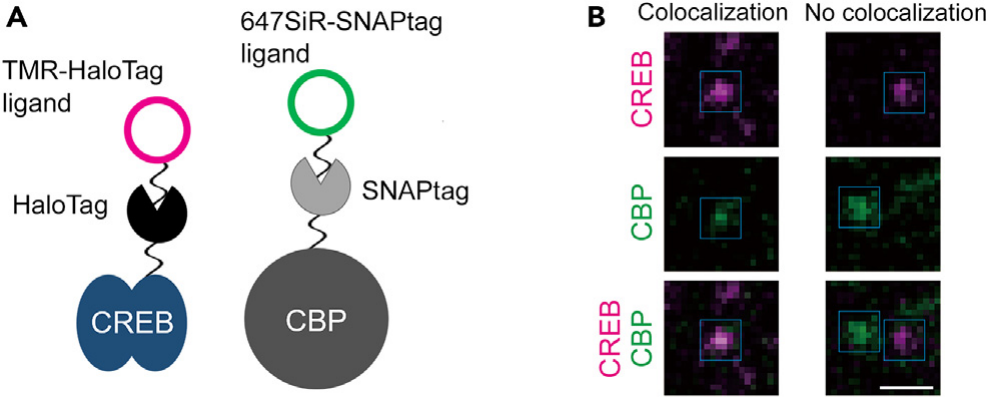
Simultaneous SMI of two different proteins (A) Dual tag system. (B) Representative high magnification images of simultaneous SMI of CREB and CBP spots. Left panel shows colocalization of CREB (magenta) and CBP (green), and right panel shows no colocalization. Blue rectangles indicate CREB and CBP spots. Scale bar: 1 μm.

The dual-color SMI is essentially the same as SMI of a single transcription factor except that plasmids encoding fusion proteins with two different tags are transfected (see the previous section). The following protocol is designed for simultaneous SMI for CREB and CBP (Figure 6A).

48. Three to seven days before the dual-color SMI experiment, add 7.5 μg pTα1-EGFP, 20 μg pTet-On Advance/TRE-Tight HaloTag-CREB and 70 μg pTRE-SNAP-tag-CBP to 200 μL PBS. Perform *in vitro* electroporation as described above.
49. One day before the experiment, add 5 μL of 10-fold diluted Dox solution to 1 mL culture medium (final concentration: 50 ng/mL).
50. Prepare 1 mL of DMEM/F-12 containing TMR-HaloTag ligand (10 nM) and 647SiR-SNAP-tag ligand (10 nM).
51. Replace the culture medium with this solution and incubate the culture for 30 min before observation. Keep the original culture medium.

***Note:*** The incubation time is increased to 30 min for labeling reaction of SNAP-tag.

52. Finally remove the ligand-containing medium and return to the original medium.
53. Switch from bypass mode to W-VIEW mode in image splitting module. Set the W-VIEW GEMINI with the dichroic mirror holder, bandpass filter, and correction lens.
54. Set the software to obtain split images using an EMCCD camera. W-VIEW GEMINI is controlled by NIS Elements.
  a. Open the NIS Elements software and select “Andor” (instead of “Roper Scientific”).
  b. Set simultaneous imaging mode in NIS elements (Optical configuration > checkmark the ‘Dual/Quad View’ Active).
55. Before observation, correct chromatic aberration manually using 0.1 μm TetraSpeck Fluorescent Microspheres.
  a. Place a microsphere-containing drop on a glass bottom culture dish.
  b. Place one drop of the immersion oil on 100x oil-immersion objective lens and mount the dish on the stage top incubator.
  c. Click the “Live” in NIS elements and excite fluorescent microspheres by HILO illumination with 561 nm and 639 nm lasers simultaneously.
  d. Search for microspheres in “Live” mode of NIS element using joystick a fine or extra fine mode and adjust the focus.
  e. Zoom in on some microspheres as much as possible to correct chromatic aberration effectively.
  f. Adjust “Alignment X, Y” in the image splitting module so that two different color spots completely overlap.
56. Activate all instruments for live cell imaging (step 29a).
57. Place one drop of the immersion oil on 100x oil-immersion objective lens again.
58. Mount the sample on the stage top incubator maintained at 37°C in a humidified atmosphere.
59. Click the “Live” button in NIS elements and excite both fluorescent dyes (TMR and 647SiR) by simultaneous HILO illumination with 561 nm and 639 nm lasers.
60. Find the cells exhibiting both TMR-CREB and 647SiR-CBP spots with a fine or extra fine mode and adjust the focus.

***Note:*** Although you cannot see EGFP signals on W-VIEW mode in NIS elements because of bypass filters in the image splitting module, you can find the signals through the eyepiece. When you search around EGFP-positive cells, you will find the cells exhibiting both TMR-CREB and 647SiR-CBP spots.

61. Fine-tune the illumination angle of the movable mirrors and the laser power manually to attain good image quality.
62. Set the parameters of the camera setting on NIS elements as below.
  a. Exposure time: 0.1 s.
  b. Gain: 300.
  c. Duration: 1–2 min.
  d. Interval: No delay.
63. Capture the series of images by clicking the “Run now” button in NIS element software (Figure 6B). To reduce focus drift during capture, we recommend that you use the Nikon Perfect Focus Systems in an inverted microscope.
64. After imaging, turn off the HILO illumination and save all imaging data in ND format. You can occasionally obtain additional images in the same cell, as in the case of single-molecule imaging (see above).
65. Repeat steps 59–64.

## Expected outcomes

A huge number of fluorescence spots (5,000 to 10,000) will be obtained from a single nucleus of cortical cells over the observation period (2 min). The above quantitative analysis demonstrates that the residence time distribution of spots can be expressed as the sum of the two exponential curves with short and long time constants (Equation 1). In the case of CREB, the long and short residence components are thought to represent specific and non-specific binding to DNA, respectively. This view can be confirmed by comparing A2/A1 between wild-type and the mutant CREB which cannot bind to DNA.^1^^,^^8^ SMI further provides spatial information of the transcription factor binding in the nucleus: Neuronal activity-dependent CREB repetitive binding can be observed at some specific nuclear locations.^1^^,^^8^ In addition, the colocalization analysis suggests the possible interactions between two molecules. Thus, the temporal and spatial properties of nuclear proteins could be revealed.

We have described the culture method of human NSCs optimized for SMI analysis, which is applicable to many cell types. Here we also introduce the analytical methods for SMI of a single and two different nuclear proteins. The present SMI can also be utilized to simultaneously study protein dynamics with conventional imaging of fluorescent proteins.^1^

## Quantification and statistical analysis

**Timing: 1–2 days**

ImageJ and ImageJ plugin, Particle Tracking Analysis (PTA), which detects and tracks each fluorescence spot automatically by centroid and 2D Gaussian fitting, enables to analyze the spatiotemporal properties of single-molecule spots (e.g., dwell time, spatial distribution). Origin 9.1 software (OriginLab) and Excel are used for data fitting and statistical analyses.

### Analysis of residence time distribution of CREB localizations

**Figure 7:**
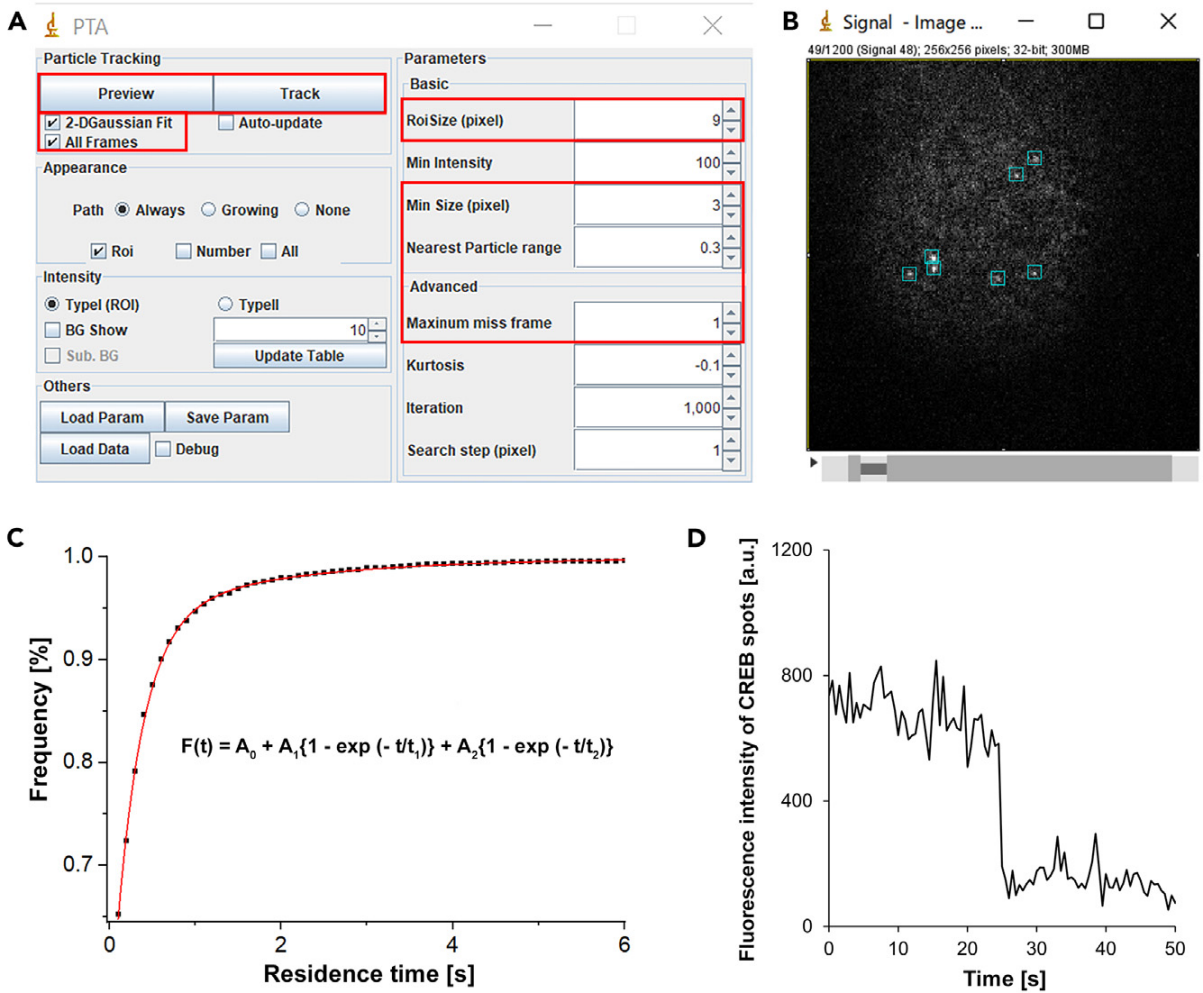
Analysis of SMI with ImageJ plugin software, PTA (A) Parameter setting in PTA. (B) Representative image processed by PTA. (C) The cumulative residence time distribution of CREB spots was fitted by the sum of two exponential curves (Equation 1). (D) Most HaloTag-CREB spots exhibited one-stepwise reduction of the fluorescence intensities by photobleaching after fixation.

***Note:*** It is important to confirm whether PTA works appropriately by checking the correspondence of CREB spots with the rectangles displayed by PTA (see Methods video S1 right).

1. Import a time series image (SIF file obtained from Solis software) containing the fluorescence intensity of an area (256 × 256 pixels) into ImageJ using “Plugins > Andor > Read SIF”.
2. The number of consecutive images can be selected. Otherwise, 1200 images taken during 2 min of observation time will be retrieved.
3. Subtract the background level from these images using ImageJ “Process > Subtract Background”. An appropriate pixel value can be selected to reduce the background levels. The subtraction is not necessary if the original images are clear.
4. Chose “Plugins > PTA > PTA Particle Track and Analysis (ver.1)”
5. Run the plugin PTA with the following parameters to detect single CREB spots (Figures 7A and 7B).
  a. Detection methods: 2 dimensional Gaussian distribution fitting.
  b. Roi size (x, y) of searching area: 9 × 9 pixels.
  c. Pixel size of minimal spots: 3 pixels.
  d. Nearest Particle range: 0.3 pixel.
  e. Maximum miss frame: 1 frame.
  f. Threshold: MaxEntropy.
  g. Other parameters are the same as the default.

6. The result table shows several parameters including the center coordinate and dwell time of each spot. Import these parameters into an Excel table.
7. Open Origin 9.1 software (OriginLab).
8. Plot the cumulative residence time distribution from the obtained data, and fit distribution with the following biexponential function curves (Figure 7C).

(Equation 1) $$F\left(t\right)=A_{0}+A_{1}\;\left\{1-\exp\left(-t/t_{1}\right)\right\}+A_{2}\;\left\{1-\exp\left(-t/t_{2}\right)\right\}$$

*t* is time and *A*_*0*_ is constant*. A*_*1*_ and *A*_*2*_ are the fractions with dissociation rate constants *1/t*_*1*_ and *1/t*_*2*_ for the short- and long-residence components, respectively.

***Note:*** To determine the time which separates the long and short residence components, the time can be determined such that the ratio of the short residence component, *A*_1_ exp (− *t*/*t*_1_)/ (*A*_0_ + *A*_1_ exp (− *t*/*t*_1_) + A_2_ exp (− *t*/*t*_2_)), is sufficiently small (for example less than 5%). It is efficient to compare A_1_ and A_2_ values with those obtained from an experiment using the mutant CREB that cannot bind to DNA. Check the validity of the fitting and evaluate which equation best fits your experiments. In our case, the chi-square tolerance for fitting with Equation 1 was less than 1 × 10^–9^. To date, the power-low model is also often used.^11^

### Analysis of simultaneous SMI of CREB and CBP localization and their colocalization

To determine colocalization of CREB and CBP, fluctuations of CREB and CBP spots are necessary to be measured. When the distance between the center coordinates of CREB and CBP spots at a given time is smaller than the sum of the localization errors of CREB and CBP spots, these spots are defined as colocalized.

9. Import the data of TMR-CREB and SNAP-CBP (ND2 file) into ImageJ.
10. These images for CREB and CBP spots can be opened as separate files.
11. First, apply CREB images to PTA as described above.
12. The result table shows each spot data including XY coordinate.
13. Relist the data based on the dwell time (frame length).
14. Focus on long residence CREB spots (for example, the dwell time > 1 s).
15. Highlight a row of each long residence CREB spot, and select “Statistics > show Statics”.
16. Another data window shows the statistics of the highlighted spot (pointindex, total frame, run length, averageX, sdX, averageY, sdY, averageFI, and sdFI).
17. Keep the values of averageX, sdX, averageY and sdY for all long residence CREB spots.
18. Determine the center coordinate of each long residence CREB spot as averageX and averageY.
19. Determine the localization error as a circle with a radius of the larger value of 1.96∗sdX or 1.96∗sdY. This indicates that the long residence CREB is considered to be present within the circle with a probability of 95% or greater.
20. Similarly, determine the XY coordinates and its localization errors for long residence CBP spots.
21. If a CREB circle overlaps with a CBP circle at the time points when both CREB and CBP spots emerged, define these CREB and CBP spots as colocalized (Figure 6B).

## Limitations

The 2-min observation is sufficient to study the transcription factor dynamics on the order of a few seconds and to examine the effects of brief or pulsed stimulation. For instance, changes of CREB dynamics after 5 min of high-frequency optogenetic stimulation can be detected in the same cell.^1^^,^^8^ However, it is difficult to examine changes that may last longer than several hours.

As for colocalization, the result simply indicates that two factors have a statistically high probability of being present in the same location, and does not necessarily show binding of these factors.

## Troubleshooting

### Problem 1

Too much cell death occurs after electroporation.

### Potential solution

Reduce the plasmid concentrations. Search for more adequate pulse duration and amplitudes. Transfection regents can be also used (e.g., NeuroMag and the magnetic plate, OZ Biosciences) to reduce cell death.

### Problem 2

Single-molecule CREB spots cannot be found under HILO illumination.

### Potential solution

Search for cells expressing fluorescent-tagged CREB spots clearly since the expression level of CREB spots varies among cells. If you can find only cells with few or too many fluorescent-tagged CREB spots, optimize the condition of *in vitro* electroporation (plasmid concentration, ratio), Dox and ligand concentrations for best imaging. For example, when you find only 1 out of 5 EGFP-positive cells in which CREB spots appear, reoptimize the aforementioned.

### Problem 3

SMI spots are difficult to detect because of low signal to noise ratio.

### Potential solution

If fluorescent-tagged CREB spots are not clear, increase exposure time, electron multiplier gain level or laser power. For better imaging, adjusting HILO illumination, the focus, and cell localization in a given field of view is important.

## Resource availability

### Lead contact

Further information and requests for resources and reagents should be directed to and will be fulfilled by the lead contact, Noriyuki Sugo (sugo@fbs.osaka-u.ac.jp).

### Technical contact

Questions about the technical specifics of performing the protocol should be directed to and will be answered by the technical contact, Yuri Atsumi (atsumi.fbs@osaka-u.ac.jp).

### Materials availability

This study did not generate new unique reagents.

### Data and code availability

This study did not generate original code and dataset.

## Acknowledgments

This work was supported by MEXT KAKENHI on Dynamic regulation of brain function by Scrap & Build system (no. JP16H06460) to N.Y, JSPS KAKENHI grants (no. JP19H03325 to N.Y. and no. JP17K071090 to N.S.), and JST SPRING (no. JPMJSP2138) to Y.A.

## Author contributions

Y.A. contributed to most descriptions of all experiments, N.Y. to the culture method, and N.S. to the SMI setup. Y.A., N.Y., and N.S. contributed to the general descriptions.

## Declaration of interests

The authors declare no competing interests.
